# Oxysterols in Autoimmunity

**DOI:** 10.3390/ijms20184522

**Published:** 2019-09-12

**Authors:** Donovan Duc, Solenne Vigne, Caroline Pot

**Affiliations:** Laboratories of Neuroimmunology, Neuroscience Research Center and Division of Neurology, Department of Clinical Neurosciences, Lausanne University Hospital and Lausanne University, Chemin des Boveresses 155, 1066 Epalinges, Switzerland; Donovan.Duc@chuv.ch (D.D.); Solenne.Vigne@chuv.ch (S.V.)

**Keywords:** Liver X receptors, oxysterols, Ebi2, ROR, Ch25h, autoimmunity, multiple sclerosis, inflammatory bowel disease

## Abstract

Cholesterol is a member of the sterol family that plays essential roles in biological processes, including cell membrane stability and myelin formation. Cholesterol can be metabolized into several molecules including bile acids, hormones, and oxysterols. Studies from the last few decades have demonstrated that oxysterols are not only active metabolites but are further involved in the modulation of immune responses. Liver X Receptors (LXRs), nuclear receptors for oxysterols, are important for cholesterol homeostasis and regulation of inflammatory response but are still poorly characterized during autoimmune diseases. Here we review the current knowledge about the role of oxysterols during autoimmune conditions and focus on the implication of LXR-dependent and LXR-independent pathways. We further highlight the importance of these pathways in particular during central nervous system (CNS) autoimmunity and inflammatory bowel diseases (IBD) in both experimental models and human studies. Finally, we discuss our vision about future applications and research on oxysterols related to autoimmunity.

## 1. Introduction

Cholesterol is implicated in several biochemistry processes of the body. It is an essential component of the mammalian cells accounting for up to 25% of all membrane lipids [1]. Its rigid hydrophobic structure confers stability on the plasma membrane and hampers the movement of other molecules, thus modifying the proportion of the cholesterol in the cell membrane can influence membrane fluidity [2]. In addition, cholesterol can interact with integral membrane proteins and modulate their functions [1]. It is also a precursor of important molecules such as vitamin D, bile acids, steroid hormones, and oxysterols.

Oxysterols are downstream metabolites of cholesterol oxidation. They can be divided into two categories called primary and secondary oxysterols. The primary oxysterols, synthesized directly from the cholesterol, are composed of side-chain oxysterols and ring-modified oxysterols. Side-chain oxysterol family includes 24S-, 25-, (25R)-26- (the latest was previously named 27- [3]), hydroxycholesterol (-OHC), and ring-modified oxysterol, which includes 7α- and 7β-OHC and 7-ketocholesterol (-KC). The secondary oxysterols, including 7α,25-dihydroxycholesterol and 7α (25R)-26-dihydroxycholesterol are generated from primary oxysterols 25-OHC and (25R)-26-OHC, respectively. Oxysterols can be synthesized via enzymatic and non-enzymatic reactions. Specific hydroxylases are responsible for enzymatic oxidation, while reactive oxygen species oxidation is mainly responsible for non-enzymatic generation of oxysterols [4].

Research on oxysterols started in the early 1940′s with studies on cholesterol autoxidation leading to the generation of oxysterols [5,6]. Growing interest in studying oxysterols continued in the late 1970 when Kandutsch and colleagues observed that oxygenated derivatives of cholesterol were able to downregulate the synthesis of cholesterol [7,8,9,10]. During the following years, several studies highlighted the importance of these molecules in a multitude of other biological processes [11]. Indeed, oxysterols were first described as a mediator of cholesterol metabolism. Oxysterols modulate the level of cholesterol intracellularly through transcriptional regulators like the liver X receptor (LXR) and the sterol regulatory element binding protein (SREBP). LXR mediates the expression of ATP binding cassette (ABC) transporter intervening in cholesterol transport and efflux [12]. SREBP also regulates the cholesterol metabolism in the cell by inducing the synthesis (through 3-hydroxy-3-methylglutaryl coenzyme A synthase/reductase) or the uptake of cholesterol (though expression of low-density lipoprotein receptor). In addition to the modulation of cholesterol levels, oxysterols are precursors of bile acid production and steroid hormones acting as intermediates in their synthesis. In the last decade, oxysterols have been proposed to act as fine-tuners of the immune responses, including trafficking of immune cells, anti-viral actions, cytokine secretions, and inflammasome modulations. In this review, we will focus on oxysterols and their downstream pathways that are implicated in immunological processes. We will further discuss their implications during autoimmune diseases.

## 2. Oxysterols: LXR Agonists and Beyond

### 2.1. LXR

**Figure 1:**
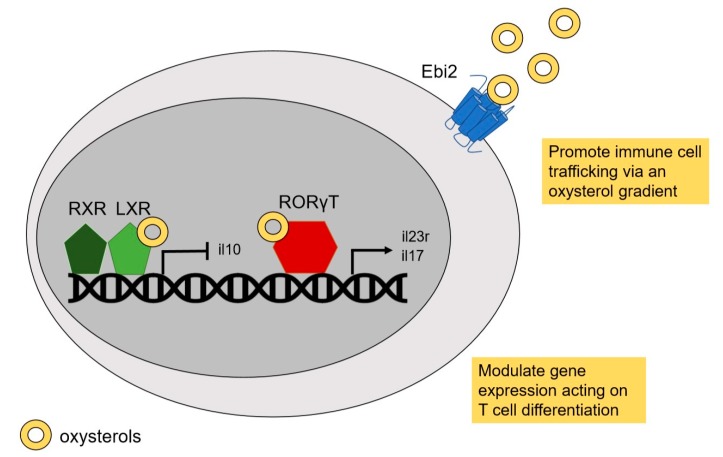
Molecular targets of oxysterol implicated in autoimmunity. Oxysterols have different targets during autoimmune diseases. Oxysterols promote immune cell trafficking through Ebi2 receptor expressed on cell surface. Oxysterol-Ebi2 interaction allows the cells to migrate via an oxysterol-gradient dependent manner. The liver X receptor (LXR) and RORγT are members of the nuclear receptors’ family of transcription factors involved in immune cell differentiation. Through those transcription factors, oxysterols modulate the gene expression implicated in inflammatory and autoimmune processes.

Different oxysterol subsets have been discovered. They are all sharing close structural similarities but have various targets and actions (Figure 1). One receptor shared by oxysterols is the LXR receptor. Side-chain oxysterol family such as 25-OHC and (25R)-26-OHC are well characterized as LXR ligands [13]. LXRs are part of the nuclear receptors’ family of transcription factors. LXRα (NR1H3) and LXRβ (NR1H2) are two isoforms that have been identified [14]. Despite the close homology between the two isoforms (almost 80% identity of their amino acid sequences are identical) [15], they are not sharing the same function nor the same pattern of expression (https://www.nursa.org, last accessed date: 8 August 2019). LXRα is expressed mostly in metabolically active tissues like liver, gut, and adipose tissue. Indeed, LXRα has been suggested to be the major sensor of dietary cholesterol. LXRβ is ubiquitously expressed. For both LXRα and LXRβ, the active form is a heterodimer composed by the association of one protein of LXR and one protein of retinoid x receptor (RXR) [16]. The heterodimer binds LXR response elements, consisting of a direct repeat spaced by 4 nucleotides [17]. LXR modulates gene expression through direct activation, repression, and transrepression [18]. At the physiological stage, LXR are important to control metabolic processes, including cholesterol homeostasis. The metabolism of cholesterol leading to oxidized cholesterol derivates is known to activate a LXR downstream pathway [19]. This process leads to an active feedback loop characterized by the activation of several genes that can modulate cholesterol levels such as cholesterol transporters (ABC transport genes) [20]. Moreover, LXRs are essential for hepatic functions and participate in the bile acid formation and control of hepatic lipogenesis. LXRs have also been characterized as important immunological modulators. LXRs are able to suppress inflammatory response through trans-repression [21]. Indeed, sumyolation of active LXR form can dampen the activity of nuclear factor κb and activator protein 1 that controls proinflammatory genes expression [22,23].

In innate immunity, LXR pathways participate in the clearance of bacteria during infection in macrophages. Indeed, induction of LXRα (but not LXRβ) expression occurs during intracellular bacterial infection [24]. Using another model of intracellular bacterial infection, Matalonga et al. have discovered that LXR activation induces cytoskeletal changes during infection by modulating nicotinamide adenine dinucleotide levels [25]. Regarding the adaptive immune system, LXRs have been described to decrease the proliferation of both T and B cells [26]. LXRβ is expressed in macrophage, T, and B cells. On the other hand, LXRα is highly expressed in peritoneal-derived and bone-marrow derived macrophages, but not in T cells or B cells. Mice lacking LXRβ show lymphoid hyperplasia and have improved responses to antigenic challenge [26]. These results were only found in LXRβ knockout but not in LXRα knockout. In addition, LXR activation inhibit IL-2- and IL-7-induced human T cell proliferation [27]. Regarding subtypes of T cells, LXRs are involved in the polarization of Th17 cells [28], subset of T helper cells that are important in autoimmune disease. Indeed, Th17 induction is facilitated in LXR knockout mice and LXR deficiency promotes Th17 polarization in vitro [28]. Finally, we demonstrated that LXRs further acts on regulatory T cells. In our study, we observed that 25-OHC, through LXR pathway, acts as a negative regulator of IL-10 secretion in murine IL-27-induced Treg [29]. Similarly, 25-OHC has been shown to down-regulate IL-10 production from human Th1 cells [30], thus highlighting a pro-inflammatory role of 25-OHC in fine-tuning CD4+ T cell polarization in different T cell subsets.

### 2.2. Retinoic Acid Receptor-Related Orphan Receptor (RORs)

Like LXRs, RORs are members of the nuclear receptor family of transcription factors binding oxysterols. They are composed of three different forms; RORα (NR1F1), RORβ (NR1F2), and RORγ (NR1F3). RORs recognize and bind as monomer to specific ROR response elements on DNA [31]. After their activation, RORs recruit co-activator and activate gene transcription [32]. Several oxysterols (i.e., 25-OHC, (25R)-26-OHC, 7α-OHC) can bind RORα and RORγ, however no study reported oxysterols as ligand of RORβ [4]. RORα is expressed in several tissues and participate in circadian rhythms, glucose and lipid metabolism, and during the development. RORγ is also expressed in multiple organs and is an important transcription factor for immune cells. RORγ has also variant including RORγ1 and RORγ2 (also known as RORγT) isoforms [33]. RORγT is an essential transcription factor in Th17 cell development [34] and drives autoimmune diseases, as will be discussed in the following chapters.

### 2.3. Epstein-Barr Virus-Induced G-Protein Coupled Receptor 2 (Ebi2)

G-protein coupled receptor 183 also known as Epstein-Barr virus-induced G-protein coupled receptor 2 (Ebi2) is a membrane receptor from the G-protein-coupled receptors (GPCR) family. Ebi2 was first discovered in Burkitt’s lymphoma cells after Epstein-Barr virus infection [35]. It was first observed in B cells but further studies have demonstrated that it was also expressed in other type of cells such as T lymphocytes, monocytes, dendritic cells, astrocytes and innate lymphoid cells. Among the different signaling pathways of GPCR, Ebi2 receptor is defined as chemotactic receptor and participates in the migratory capability of cells [36,37,38]. The most potent endogenous ligand of this membrane receptor is the oxysterol 7α-25-OHC produced from oxidation of 25-OHC by Cyp7b1 enzyme [38,39]. Cells that express Ebi2 are trafficking through an oxysterol gradient dependent manner acting like chemokine processes (Figure 1). The migratory function of this receptor affects several important immune processes. In particular, Ebi2 is involved in the T-dependent antibody response in the germinal centers [37]. Indeed mice lacking Ebi2 have an abnormal positioning of B cells in the follicular regions of secondary lymphoid organs [39]. A recent study demonstrated that Ebi2 drives CD4+ T cells peripheralization in lymph node [40]. Mice lacking Ebi2 receptor have a CD4+ T cells location issue and have delayed responses in antigen recognition and proliferation in the lymph node.

## 3. Oxysterols in CNS Autoimmunity

### 3.1. Oxysterols

As growing evidence supported roles in immune regulation involving oxysterols, the scientific community further studied the role of cholesterol metabolites in autoimmune conditions. Multiple sclerosis (MS) is the most common autoimmune disease involving the nervous system [41]. MS and its animal model, the experimental autoimmune encephalomyelitis (EAE), are characterized by inflammatory cell infiltrates and demyelination in the central nervous system (CNS), leading to neurological damage. A combination of both genetic and environmental factors has been proposed to trigger the disease. In this vein, obesity has been described as a risk factor for MS. Indeed, a direct correlation between a higher body mass index during childhood [42,43] or adolescence [44,45] and a higher risk of developing MS has been observed in several epidemiological studies. In addition, metabolic changes linked with obesity such as an altered lipid profile are associated with poor outcome of MS [46,47,48,49,50]. Moreover, obesity and high fat diet have been associated with perturbation of cholesterol and oxysterol homeostasis in the liver, hypothalamus, adipose tissue and plasma in an experimental model [51].

Oxysterol perturbations have been further described in MS. First, it has been proposed that MS patients have disrupted oxysterol levels compared to healthy controls both in blood and in cerebrospinal fluid (CSF). In particular the plasma levels of 24-OHC, (25R)-26-OHC, and 7α-OHC were significantly lower in MS patients compared to in healthy control [52] and in the CSF of MS patients, a reduction of the concentration of 25-OHC and (25R)-26-OHC was observed [53]. Similarly, an evaluation of oxysterol levels was performed in MS patients and controls in a longitudinal study (5 year) and significant modulations of circulating oxysterols were observed in MS patients but not in controls: 24-OHC, (25R)-26-OHC, and 7α-OHC levels were lower in MS patients compared with healthy controls, and 7-KC was higher in progressive MS compared with relapsing-remitting MS [54]. However, while a decreased 24-OHC-blood level was observed with advanced Alzheimer’s disease as well as with MS disease, increased 24-OHC-blood levels have been observed in early neurodegenerative processes in both diseases [55]. 24-OHC is the predominant metabolite of brain cholesterol [56] and several studies reported a modulation of 24-OHC in the CSF and serum of MS patients [57]. Interestingly, the disease-modifying therapy natalizumab, reduces the concentrations of 24-OHC and (25R)-26-OHC in CSF [58]. As 24-OHC indicates CNS cholesterol turnover [59,60] and has been proposed as a biomarker for neurodegeneration [61] and for clinical stages of MS [62]. It has been proposed that a decrease of 24-OHC after natalizumab treatment might reflect reduced neuronal damage. Regarding (25R)-26-OHC, the majority of this oxysterol in the CSF is coming from peripheral blood and the concentration depends on the blood brain barrier (BBB) integrity [58]. As natalizumab acts on the BBB functionality [63], the authors hypothesized that reduction of (25R)-26-OHC in the CSF could be associated with an improvement of BBB integrity.

Genetic analysis of MS patients further revealed a potential association between genetic variants of cholesterol 25-hydroxylase (Ch25h) and primary progressive MS patients, supporting a role for Ch25h and related-oxysterols in CNS autoimmunity [64]. Moreover, genetic variants in NR1H3 (LXRα) were also found to be associated with increased risk of developing progressive MS [65,66]. These recent studies on human strongly suggest that perturbation of oxysterol metabolism may influence the progression of MS disease. However, the underlying mechanisms are still unclear and several research groups, including ours, are working on understanding the role of oxysterols in CNS autoimmunity using experimental models.

### 3.2. LXR

In 2006, Hindinger et al. published the first evidence for a role of LXR in the EAE model. Using the LXR agonist ligand T0901317, they observed that it reduced EAE clinical severity and CNS inflammation [67]. Additional studies found that in vivo administration of LXR agonists decreased IL-17 secretion [28] and suppressed IL-17A, IFNγ, and IL-23R expression [68]. As Th17 cells largely contribute to EAE development [69,70], these results are associated with the dampened EAE severity observed [68]. Moreover, both mice and human Th17 cells were downregulated by LXR activation [28]. Th17 cell differentiation is controlled by LXR through the activation of Srebp-1a and Srebp-1c. Overexpression of SREBF-1a and SREBF-1c dampened the differentiation of Th17 cells by physically interfering with the Ahr transcription factor and inhibiting Ahr-controlled IL-17 transcription [28]. In contrast, knocking down of either SREBF1 isoforms resulted in an increase of Th17 cell differentiation [28]. Interestingly, LXR/RXR pathways and SREBF1 modulate encephalitogenic Th17 cells during the adoptive transferred EAE. The authors compared the transcriptome transition of encephalitogenic Th17 cell before (in vitro) and after adoptive transfer in the CNS of recipient mice [71]. LXR/RXR and downstream target genes, including genes important for cholesterol transport such as Lpl, Abca1, and Abcg1 were found to be increased in Th17 cells during EAE compared to Th17 differentiated in vitro. In contrast, SREBF1, which controls the expression of genes involved in fatty acid and triglycerides synthesis, was found to be downregulated in Th17 cells located in CNS of EAE mice compared to in vitro differentiated Th17 cells [71]. Even if the precise role of LXR pathway in CNS autoimmunity remains to be further investigated, modulation of the LXR pathway and their target genes are involved in a metabolic checkpoint during Th17 cell differentiation which is important in MS and EAE diseases [71]. Beyond the regulation of T cells, LXR pathways influence other types of CNS cell population. LXR activation via oxysterols downregulated pro-inflammatory responses in microglial and astrocytes in vitro. As EAE and MS also involved glial cells, it could further explain the role of LXR activation in the development of these diseases [72,73,74] Moreover, LXRα has been shown to modulate the BBB permeability and to affect EAE severity. Using mice with specific depletion of LXRα in endothelial cells, the authors observed a worsened EAE disease compared to controls [75].

### 3.3. ROR

As introduced above, RORγT, a target for several oxysterols, is an essential transcription factor for Th17 cell differentiation [76]. As EAE is mediated mainly by Th17 cells, several reports have studied the role of this transcription factor in CNS autoimmunity. RORγT knockout mice are less susceptible to EAE disease and depict a reduction of Th17 cell infiltration in the CNS [34]. In contrast, overexpression of RORγT led to increased EAE disease severity [77]. As lymph nodes are absent in RORγT knockout mice [78], it is difficult to decipher whether the therapeutic potential of RORγT in reducing EAE is secondary to the lack of RORγT in the T cells, or to the lack of lymph nodes. However, Yang et al. found that suppressing RORγT expression specifically in encephalitogenic T cells did not reduced EAE disease using adoptive transferred EAE [79]. Additional study demonstrated that RORα is also involved in the differentiation of Th17. Mice knockout for RORα have reduced level of IL-17 production and develop milder clinical symptoms during EAE disease. RORα and RORγT synergized in promoting Th17 differentiation. Moreover, double deficiencies in RORα and RORγ completely impaired Th17 generation in vitro and fully protected mice from EAE development [80].

### 3.4. Ebi2

**Figure 2:**
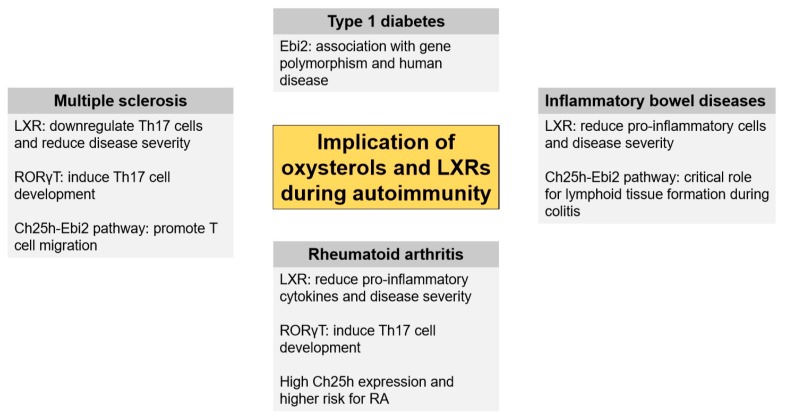
Implications of oxysterols and LXRs in autoimmunity. The roles of oxysterols and LXR-dependent and LXR-independent pathways have been studied in different autoimmune conditions.

Ebi2 is involved in migration of immune cells. As T cell trafficking plays a major role in MS and EAE, Ebi2 and related oxysterols have been studied recently in this context (Figure 2). In our laboratory, we demonstrated that oxysterols regulate the trafficking of encephalitogenic T cells during the development of EAE disease [81]. Indeed, Ch25h-deficient mice show an attenuated EAE disease course by limiting the trafficking of pathogenic Th17 lymphocytes to the CNS. We further observed an accumulation of Th17 lymphocytes in the peripheral lymph nodes in the absence of Ch25h-related oxysterols during EAE, thus pointing towards a possible defect in T lymphocytes exit from the lymph nodes. Interestingly, this is reminiscent of the fingolimod mechanism of action, a drug that constrains MS inflammatory activity by trapping a subset of the T cell in the lymph nodes [82]. We further observed that T lymphocytes migrate specifically in response to 7α,25-OHC through Ebi2 signaling. Independently, other authors reported that Ch25h and Cyp7b1 expression as well as 7α,25-OHC level were increased in CNS during EAE development [83]. They further proposed that Ebi2 is predominantly expressed in Th17 cell subset compared to Th1 or CD8+ T cells and that its expression is maintained by pro-inflammatory cytokines (i.e., interleukin-23 and interleukin-1β). The capacity of Ebi2^-/-^ Th17 cells to induce CNS autoimmunity was established using an adoptive transfer model of EAE. Mice that received encephalitogenic Ebi2^-/-^ Th17 cells had a delayed disease development compared to mice transferred with wild-type controls [83]. In addition, inflamed white matter of MS patients showed a high expression of Ebi2 receptor compared to the non-inflamed region of the white matter and a proportion of T cell expression Ebi2 was described in the lesions of MS patients [83]. Moreover, we characterized the Ebi2 expression profile in human lymphocytes in MS patients and observed that Ebi2 is functionally expressed on memory CD4+ T cells [84]. Interaction between Ebi2 receptor and oxysterols fine-tunes immune cell migration, a mechanism used by several treatments for MS, such as natalizumab, which blocks the entry of immune cells into the CNS [63]. Interestingly, memory CD4+ T cells from MS patients treated with natalizumab display an increased Ebi2 expression and migration profile to 7α,25-OHC, suggesting an important role for Ebi2 and related oxysterol in human CD4 T cell migration in MS patients [84]. Finally, oxysterol levels are altered in the CNS during EAE development. Among the several oxysterols found in the CNS, 7α,25-OHC is significantly increased during EAE and could potentially be associated with the increase immune cell infiltrates observed during the disease [85]. However, the precise role of 7α,25-OHC and its exact cellular source in the CNS during EAE remain still unknown.

## 4. Oxysterols in Inflammatory Bowel Disease (IBD)

### 4.1. Oxysterols

Inflammatory bowel disease (IBD) regroup two frequent chronic diseases of the gut: the ulcerative colitis (UC) and Crohn’s disease (CD). Several examples of evidence showing a relation between oxysterols and inflammatory disorders such as IBD have appeared throughout the last years. Oxysterols which originated from diet are totally absorbed by the gut, which represents the initial site of exposure to their effects. They are suggested to potentially interfere with homeostasis of the human digestive tract, playing a role in intestinal mucosal damage. Oxysterols were found most commonly in cholesterol-rich food as a mixture [86,87,88]. Several in vitro studies proposed that a mixture of oxysterols derived from dietary cholesterol led to a strong pro-inflammatory effect and exhibited cytotoxicity, apoptosis, and development of atypical cell clones of human colonic epithelial cells, favoring in vitro intestinal inflammation and colon cancer progression [89,90,91,92,93,94,95]. In addition, oxysterols such as 7-KC and 25-OHC were also described to decrease the barrier integrity of vascular endothelium and intestinal epithelial [96]. These in vitro studies on intestinal cells suggest that oxysterols are able to interfere in different steps of colonic inflammation (Figure 2). Finally, intestinal fibrosis and stenosis are common complications of CD that do not respond to anti-inflammatory treatments. Interestingly, oxysterols downstream Ch25h enzyme are further implicated in the pathogenesis of intestinal fibrosis and could thus contribute to IBD on several aspect of the disease [97].

### 4.2. LXR

In the colon of human and mice, both LXR subtypes are expressed and were reported to have anti-inflammatory effects in colon epithelial cells [98]. In two different experimental model of IBD, it was reported that LXR-deficient mice were more susceptible to colitis with a more protective role for LXRβ than LXRα in both DSS and TNBS-induced colitis. In addition, activation of LXR receptors by synthetic ligands accelerates disease recovery in DSS-induced colitis [98]. LXR activation via oral application of LXR agonist reduced pro-inflammatory Th1 and Th17 cells while induced gut-associated regulatory T cells [99]. Polymorphisms in LXRs were shown to be associated with IBD in a Danish study and the mRNA expression for both LXRs are decreased in CD and UC patients compared to healthy controls [98,100]. Recently, two independent studies reported that both oxysterols, particularly 4β-HC and 25-OHC as well as their metabolizing enzyme levels, were altered in acute or chronic colitis models in mice and in biopsies of human colitis cohorts [101,102]. However, despite evidence showing the relation between oxysterol/LXR receptors and intestinal inflammation, it is difficult to evaluate the precise role of oxysterols and their nuclear receptors during colitis, which needs further investigation.

### 4.3. Ebi2

In 2012, Ebi2 was identified as an IBD risk gene by genome-wide association studies (GWAS) and a single nucleotide polymorphism in Ebi2 increase the risk for both, CD and UC with genome wide significance [103]. Moreover, a significant upregulation of EBI2 gene was found in the ileum of CD patients with NOD2 risk allele [104]. The role of EBI2 during intestinal inflammation was recently studied using different mice model of colitis [102,105,106]. Using an innate model of intestinal inflammation, Emgard et al. showed that Ebi2 deficient mice were less susceptible to colitis [105]. Ebi2 is highly expressed by type 3 innate lymphoid cells (ILC3s), whereas oxysterols synthetized enzymes were mostly produced by fibroblastic stromal cells found in intestinal lymphoid structures. Ebi2 and its oxysterol ligand were shown to be essential for the localization and the migration of ILC3s and to have a critical role for the formation of lymphoid tissues in the mouse colon during colitis [105,106]. The data suggest an implication of EBI2/oxysterol axis for controlling and regulating colonic lymphoid tissues organization during intestinal inflammation.

## 5. Other Autoimmune Diseases

### 5.1. Rheumatoid Arthritis (RA)

Rheumatoid arthritis (RA) is a chronic autoimmune disorder that primarily affects the joints. The disease is characterized by an infiltration of inflammatory leukocytes in the synovial compartment and autoantibodies that are also found in 50% to 70% of patients [107]. LXR have been hypothesized as a possible therapeutic target for RA. Indeed, the first study investigating the role of LXR pathways reported that LXR agonist (T0901317) reduced the clinical symptoms in the murine collagen-induced arthritis (CIA) model [108]. Similar results were found in two other studies showing that LXR ligand (GW3965) attenuated the symptoms associated with a decreased pro-inflammatory cytokines production [109,110]. Contrary results were found in which the authors observed a dose-dependent exacerbation of arthritis disease when mice were treated with two LXR ligands (T0901317 and GW3965) [111]. The authors explained these discordant findings by the different doses and routes of the drug administration used.

RORs were also found involved in the RA. RORC gene, coding for RORγ, is found to be highly expressed in the CD4+ T cell of patients with a recent RA disease compared to healthy controls [112]. Using the CIA model, one study has shown that the inverse agonist of RORγ can decrease the development of arthritis [113]. Moreover, Xue et al. found similar results using a selective inverse agonist of RORγT [114]. On the other hand, overexpression of RORγT in T cells also attenuated the arthritis in mice, however the precise mechanisms are not yet fully understood [115]. Finally 25-OHC dampens IL-10 production in Th1 cells that also contribute to the disease progression in RA. Interestingly, synovial Ch25h expression mRNA expression is highly expressed in individuals that depict autoantibody-positive arthralgia and that are at high risk of developing RA [30]. The role of oxysterols and Ch25h-pathway thus remain to be further investigated in RA (Figure 2).

### 5.2. Type 1 Diabetes (T1D)

Type 1 diabetes (T1D) is a T-cell–mediated autoimmune disease that destroys insulin-producing pancreatic β-cells. Very few studies examine the implication of oxysterol or cholesterol biosynthesis pathway during T1D. Using experimental models, Yoshioka et al. measured high levels of cholesterol oxides in the kidney, heart, and liver of diabetic rats [116]. Using mass spectrometry on human blood, one study describes increased levels of total oxysterols, particularly 7β-OH-chol, in T1D patients compared to subjects without diabetes [117]. Furthermore, in another study, T1D patients also had higher plasma oxysterol levels, more specifically plasma 7-KC and chol-triol levels compared to healthy controls [118]. In 2010, a GWAS meta-analysis of T1D using both rat and human blood have linked polymorphisms in the EBI2 gene with T1D [119]. These data suggest that oxysterols could further be implicated in T1D and could even be promising suitable biomarkers to monitor the intensity of lipid oxidative modifications during T1D. However the significance and the underlying mechanisms of oxysterol production and their biological activities in T1D remain to be elucidated (Figure 2).

## 6. Conclusions

Through work over the last several decades, it is now recognized that cholesterol metabolites, in particular oxysterols, are involved in fine-tuning the immune responses and contribute to the development of several autoimmune diseases, including MS, IBD, RA, and possibly T1D. The complexity of oxysterol downstream pathways with several intracellular nuclear factors as well as with membrane surface receptors certainly contributes to the different implications of oxysterols pathways during autoimmunity. The precise contribution of both LXR-dependent and independent pathways is still largely undetermined in this context. It thus remains a field that needs to be further investigated to fully understand how oxysterols are generated and participate to autoimmunity. By understanding the precise role of cholesterol pathways during inflammation, we can anticipate the emergence of new therapeutic treatments to tackle autoimmune diseases.

## Abbreviations

ABC	ATP binding cassette
BBB	Blood brain barrier
CD	Crohn’s disease
Ch25h	Cholesterol 25-hydroxylase
CIA	Collagen-induced arthritis
CNS	Central nervous system
CSF	Cerebrospinal fluid
EAE	Experimental autoimmune encephalomyelitis
Ebi2	Epstein-Barr virus-induced G-protein coupled receptor 2
GPCR	G-protein-coupled receptors
GWAS	Genome-wide association studies
IBD	Inflammatory bowel disease
KC	Ketocholesterol
LXR	Liver X receptor
MS	Multiple sclerosis
OHC	Hydroxycholesterol
RA	Rheumatoid arthritis
ROR	Retinoic acid receptor-related orphan receptor
RXR	Retinoid X receptor
SREBP	Sterol regulatory element binding protein
T1D	Type 1 diabetes
UC	Ulcerative colitis
